# Baseline Serum Levels of Beclin-1, but Not Inflammatory Factors, May Predict Antidepressant Treatment Response in Chinese Han Patients With MDD: A Preliminary Study

**DOI:** 10.3389/fpsyt.2019.00378

**Published:** 2019-06-06

**Authors:** Shen He, Duan Zeng, Feikang Xu, Jianping Zhang, Nan Zhao, Qiang Wang, Jiali Shi, Zhiguang Lin, Wenjuan Yu, Huafang Li

**Affiliations:** ^1^Department of Psychiatry, Shanghai Mental Health Center, Shanghai Jiao Tong University School of Medicine, Shanghai, China; ^2^Psychiatry Research, Zucker Hillside Hospital, Northwell Health System, Glen Oaks, NY, United States; ^3^Shanghai Pudong New Area Mental Health Center, Tongji University School of Medicine, Shanghai, China; ^4^Biochemistry Laboratory, Shanghai Mental Health Center, Shanghai Jiao Tong University School of Medicine, Shanghai, China; ^5^Shanghai Key Laboratory of Psychotic Disorders, Shanghai Mental Health Center, Shanghai Jiao Tong University School of Medicine, Shanghai, China; ^6^Clinical Research Center, Shanghai Jiao Tong University School of Medicine, Shanghai, China

**Keywords:** beclin-1, autophagy, major depressive disorde, treatment response, predictor

## Abstract

Currently, the choice of medical treatment for major depressive disorder (MDD) is primarily based on a trial-and-error process. Thus, identification of individual factors capable of predicting treatment response is of great clinical relevance. Recent work points towards beclin-1 and inflammatory factors as potential biomarkers of antidepressant treatment response. The primary aim of the study was to investigate whether pre-treatment serum levels of beclin-1 and inflammatory factors could predict antidepressant treatment response in Chinese Han patients with MDD. Forty patients with MDD were treated with either a selective serotonin reuptake inhibitor (SSRI) (paroxetine in 20 cases) or a serotonin–norepinephrine reuptake inhibitor (SNRI) (duloxetine in 13 cases and venlafaxine in 7 cases). Depression scores and serum levels of beclin-1 were measured at the baseline and after 8 weeks of antidepressant treatment. Serum C-reactive protein (CRP), interleukin (IL)-1B, and IL-6 levels were determined using enzyme-linked immunosorbent assay kits at the baseline. Twenty-seven patients were identified as treatment responders, whereas 13 were identified as non-responders after 8 weeks of antidepressant treatment. Baseline serum beclin-1 levels were significantly higher in non-responders than in responders (p = 0.001), whereas no differences were found in baseline serum CRP, IL-1B, or IL-6 levels between responders and non-responders. There were no significant correlations between baseline levels of beclin-1 and baseline IL-1β, IL-6, and CRP levels—neither in the total sample nor in responder and non-responder groups. Moreover, logistic regression models and a random forest model showed that baseline serum beclin-1, but not inflammatory factors, was an independent and the most important predictor for antidepressant treatment response. Furthermore, serum beclin-1 levels were significantly increased in responders (p = 0.027) but not in non-responders after 8 weeks of treatment (p = 0.221). Baseline serum beclin-1 levels may be a predictive biomarker of antidepressant response in patients with MDD. Moreover, beclin-1 may be involved in the therapeutic effect of antidepressant drugs.

## Introduction

Major depressive disorder (MDD) is a severe and debilitating illness that is estimated by the World Health Organization to affect 350 million people worldwide (1). At present, although the identification of blood biomarkers that can predict the treatment response to antidepressants is urgently needed and useful in clinical practice, no reliable predictor has been identified (2).

Autophagy is an important clearance system for cellular waste, including toxic protein aggregates (3). It is important for most cells in various tissues, including the central nervous system (4). Dysregulation of autophagy leads to accumulation of misfolded protein aggregates and damaged organelles, leading to neuronal dysfunction and even death (5, 6). Recently, it has been reported that neuronal autophagy pathways are involved in MDD and affected by antidepressants (7, 8). Beclin-1 is an important marker of autophagy because of its essential role in the early nucleation step of autophagy (9). Several studies have demonstrated that the cellular autophagy marker, beclin-1, is upregulated upon treatment with antidepressants (10, 11). Gassen et al. found beclin-1 levels were increased in the mouse brain following antidepressant treatment. In addition, importantly, a significant positive correlation was observed between baseline expression levels of beclin-1 in peripheral blood mononuclear cells (PBMCs) and clinical response after 6 weeks of antidepressant treatment in patients with depression (11). On the basis of these findings, we hypothesized that beclin-1 may be a potential biomarker to predict response to antidepressant drugs in patients with MDD. At the present time, no clinical studies have yet reported the association between serum concentration of beclin-1 and antidepressant response in MDD patients.

In addition to the autophagy pathways, many lines of evidence have implicated inflammation in the pathogenesis of depression and mechanisms of antidepressant response (12, 13). Depressed patients have been reported to have increased serum levels of some pro-inflammatory cytokines, and antidepressant treatment may normalize levels (14, 15). Moreover, several pro-inflammatory cytokines have been studied and suggested as potential biomarkers to predict antidepressant response by many researchers (16). For instance, Uher et al. found serum C-reactive protein (CRP) levels at baseline predict differential response to escitalopram [a selective serotonin reuptake inhibitor (SSRI) antidepressant] and nortriptyline (a tricyclic antidepressant) (17). Song et al. observed that responders to serotonin reuptake inhibitor (fluoxetine) treatment had lower end-treatment interleukin (IL)-1β than had non-responders (18). Yoshimura et al. reported plasma levels of IL-6 could distinguish between responders and non-responders to an SSRI trial (19, 20). Whether baseline inflammatory factors could predict antidepressant response has still not been fully investigated in Chinese Han patients. A previous study reported that serum CRP levels may predict antidepressant treatment responses in Chinese Han patients (21). However, the study was retrospective, and the researchers focused on only one inflammatory factor. In addition, some of the patients were receiving medication before they were included in their study. Thus, further studies are also warranted in Chinese patients with MDD.

Up to now, it has been suggested that the role of innate and adaptive immunity could contribute to the development of MDD (22). Moreover, inflammation, mainly cytokine production, has become an accepted part of the etiology of MDD (23). Interestingly, previous studies have found that the interplay between autophagy and pro-inflammatory cytokines may be a fundamental mechanism coordinating the activity of innate and adaptive immune systems (24). For example, interferon (IFN)-γ, tumor necrosis factor (TNF)-α, IL-1, IL-2, IL-6, and transforming growth factor-β have been shown to induce autophagy, whereas the process is blocked by IL-4, IL-10, and IL-13 (24). Moreover, autophagy can itself modulate the production and secretion of cytokines, including IL-1, IL-18, TNF-α, and type I IFN (24). Based on these study results, it appears plausible to hypothesize that autophagy may contribute to the development of MDD, at least in part, because of the bidirectional regulatory relationship between autophagy and inflammatory cytokines. A previous study has suggested that beclin-1 could serve as a linkage between autophagy and inflammation (25). In addition, some inflammatory markers have been shown to regulate autophagy and change the expression of the beclin-1 (26, 27).

However, thus far, most investigations have been performed in cellular and animal studies with little progress being made in the clinical setting. Therefore, in this study, we measured serum levels of autophagy-related marker beclin-1 and some inflammatory markers (IL-6, IL-1β, and CRP) together at baseline in 40 patients with MDD. The primary aim of the current study was to analyze whether pre-treatment serum levels of beclin-1 and inflammatory factors could predict antidepressant treatment response. As a secondary aim, we investigated whether antidepressant treatment can change serum beclin-1 levels as there are no data in this regard.

## Materials and Methods

### Participants and Clinical Assessment

The analysis of the influence of these serum factors on antidepressant response was designed as a separate add-on biomarker study to a prospective randomized cohort study on the efficacy and tolerability of the serotonin reuptake inhibitor, paroxetine, and serotonin–norepinephrine reuptake inhibitor (SNRI), venlafaxine/duloxetine. In this report, the study population was a subsample of the prospective randomized study cohort. Data on serum levels of beclin-1 and inflammatory factors were available from 40 MDD patients, because only 40 patients provided written informed consent for the biomarker study. The enrollment of *patients was consecutive*. All diagnoses were made using the Structured Clinical Interview for DSM-IV (SCID) (28–30). The inclusion criteria were as follows: 1) age 18–65 years, 2) Han ethnicity, 3) either medication naive or medication free for at least 8 weeks before recruitment, and 4) 17-item Hamilton Depression Rating Scale (HAMD-17) score ≥20. The exclusion criteria were as follows: 1) having any other psychiatric disorders other than MDD; 2) having any physical disease (i.e., organic brain disease, endocrine disease, metabolic disorder, autoimmune disease, cardiovascular disease, and cancer or infection); 3) being pregnant or breastfeeding; and 4) no alcohol or other substance abuse.

All patients underwent a clinical interview, laboratory test, and physical examination to rule out physical illnesses. The patients were treated with SSRIs (paroxetine in 20 cases) or SNRIs (duloxetine in 13 cases and venlafaxine in 7 cases) for 8 weeks. The initial dose of paroxetine, duloxetine, and venlafaxine was 20, 40, or 75 mg/day, respectively, and the dose was increased according to the clinical response and patient tolerance (the dose was not fixed). The maximum dose of paroxetine, duloxetine, and venlafaxine was 40, 60, and 225 mg/day, respectively. The HAMD-17 was used to evaluate the severity of symptoms in MDD patients at baseline (w0) and after 8 weeks of treatment (w8). The protocol of this study was approved by the Ethics Committee of the Shanghai Mental Health Center. All patients gave written informed consent for the biomarker study. We defined the patients whose HAMD-17 scores decreased by 50% or more as responders and those with a less than 50% reduction as non-responders.

### Blood Sample Collection

Following an overnight fast, 5 ml of venous blood samples was collected between 7 and 9 a.m. After collection, the blood samples were centrifuged at 3,000 rpm at 4°C for 20 min and stored at −80°C before laboratory assays. The serum samples were collected both at baseline and after 8 weeks of antidepressant treatment.

### Analysis of Serum

Serum levels of beclin-1, IL-1β, IL-6, and CRP for each sample were measured using the enzyme-linked immunosorbent assay (ELISA) method with a human beclin-1 ELISA kit (Xitang, Shanghai, China), human IL-1β ELISA kit (Catalog # EL10028-2, Anogen, Mississauga, Canada), human IL-6 ELISA kit (Catalog # EL10023-2, Anogen, Mississauga, Canada), and high-sensitivity CRP ELISA kit (Catalog # DM E-4600, LDN, Germany) according to their respective manufacturer’s protocols. The concentrations of beclin-1, IL-1β, and IL-6 are expressed as pg of protein/ml of serum, and that of CRP is expressed as ng of protein/ml of serum. The detection limits for beclin-1, IL-1β, IL-6, and CRP were 15 pg/ml, 2 pg/ml, 7 pg/ml, and 10 ng/ml, respectively. The mean intra-assay and inter-assay coefficients of variation were below 9.6%.

### Statistical Analysis

Baseline clinical characteristics were compared between the responder and non-responder groups using unpaired *t*-test for normally distributed continuous variables and the Mann–Whitney *U* test for non-normally distributed variables. The chi-square test or *continuity*-adjusted *chi*-*square test* was used for dichotomous variables, when appropriate. The Mann–Whitney *U* test was used to evaluate the significance of differences in individual serum biomarker baseline levels expressed between the responder group and the non-responder group. To investigate whether baseline serum factors level could be used to predict an antidepressant treatment response in patients, the following statistical methods were used.

First, we used the logistic regression model. The univariate logistic regression analysis was performed to examine the predictive potential of serum factors. Then, to assess any independent factors affecting the response to antidepressant treatment, multivariate logistic regression analysis was performed for variables with a statistically significant difference in the univariate logistic regression (p < 0.1). To avoid omitting significant predictors, we included some important sociodemographics and clinical characteristics and constructed several multiple forward logistic regression models. Multicollinearity was examined using collinearity diagnostic statistics. Variance inflation factor (VIF) values > 4.0 or tolerance <0.25 may indicate a concern for multicollinearity in multivariate regression models (31). The Box–Tidwell test was conducted to identify the assumption for log-linearity in continuous variables. Moreover, a receiver operating characteristic (ROC) curve was performed to identify the optimal cut-off value of the serum levels for the prediction of the therapeutic response. The optimal cut-off values were determined using the Youden index (maximum [sensitivity + specificity – 1]) (32).

Second, random forest analysis was used to sort the order of the importance of each serum peripheral biomarkers and other sociodemographic variables including age, sex, and body mass index (BMI) for the contribution to predicting antidepressant response. The Gini index is commonly used to measure the ability of a potential discriminative test of each feature that can be defined as 1 − ∑*jp* ^2^( *j*∣*t*), where *p*( *j*∣*t*) is the estimated class probability for feature *t* or node *t* in a decision tree and *j* is an output data or class. In this study, *j* = 2 is represented as responders = yes and responders = no (33). The input variables were ranked by relative importance in predicting antidepressant response based on the mean decrease in Gini (MDG) index. Variables are displayed in the variable importance plot created for the random forest by this measure. The most important variables of the model will be highest in the plot and have the largest MDG values; the implementation of the random forest model, as well as the runs of the MDG, were executed using the R package, randomForest (34).

All results are presented as the mean ± standard deviation. Statistical tests were two tailed, and a *p*-value of <0.05 was considered statistically significant. Statistical analyses were performed using SPSS 17.0 (SPSS, Inc., Chicago, IL) software and R programming language (version 3.2.2 for Windows).

## Results

### Subjects

A total of 40 subjects [15 (38%) males and 25 (62%) females, age 36.75 ± 13.00 years] were included in the analysis. After 8 weeks, all patients completed the follow-up evaluation and sample collection. Treatment for 8 weeks resulted in significant improvement of depressive symptoms as measured by HAMD-17 total scores (w0 = 22.85 ± 3.03; w8 = 9.43 ± 7.75; *Z* = −5.375, *p* < 0.001). Of all patients, 27 (67.5%) patients were responders and 13 patients were non-responders. The demographic characteristics and clinical data of responder patients and non-responder patients are listed in **Table 1**. No significant difference was found between responders and non-responders with regard to age, sex, BMI, or baseline HAMD-17 total scores.

**Table 1:** Demographic and clinical data of responders and non-responders.

	Non-responders *N* = 13	Responders *N* = 27	*Z* or *χ * ^2^	*p*-value
Age	32.15 ± 13.27	38.64 ± 12.56	−1.618	0.106^a^
Sex (male/female)	5/8	10/17	0.000	1.000^b^
BMI	23.54 ± 3.35	22.67 ± 3.32	−0.996	0.319^a^
Medication (SSRI/SNRI)	6/7	14/13	0.114	0.736^c^
Pretreatment HAMD-17 scores	22.92 ± 2.43	22.81 ± 3.32	−1.042	0.297^a^
Post-treatment HAMD-17 scores	18.00 ± 7.40	5.30 ± 3.21	−5.051	<0.001^a^

Values are presented as mean ± SD.

a Mann–Whitney U test.

b Continuity-adjusted chi-square test.

c Chi-square test.

### Non-Responders Showed Higher Baseline Serum Levels of Beclin-1

**Figure 1:**
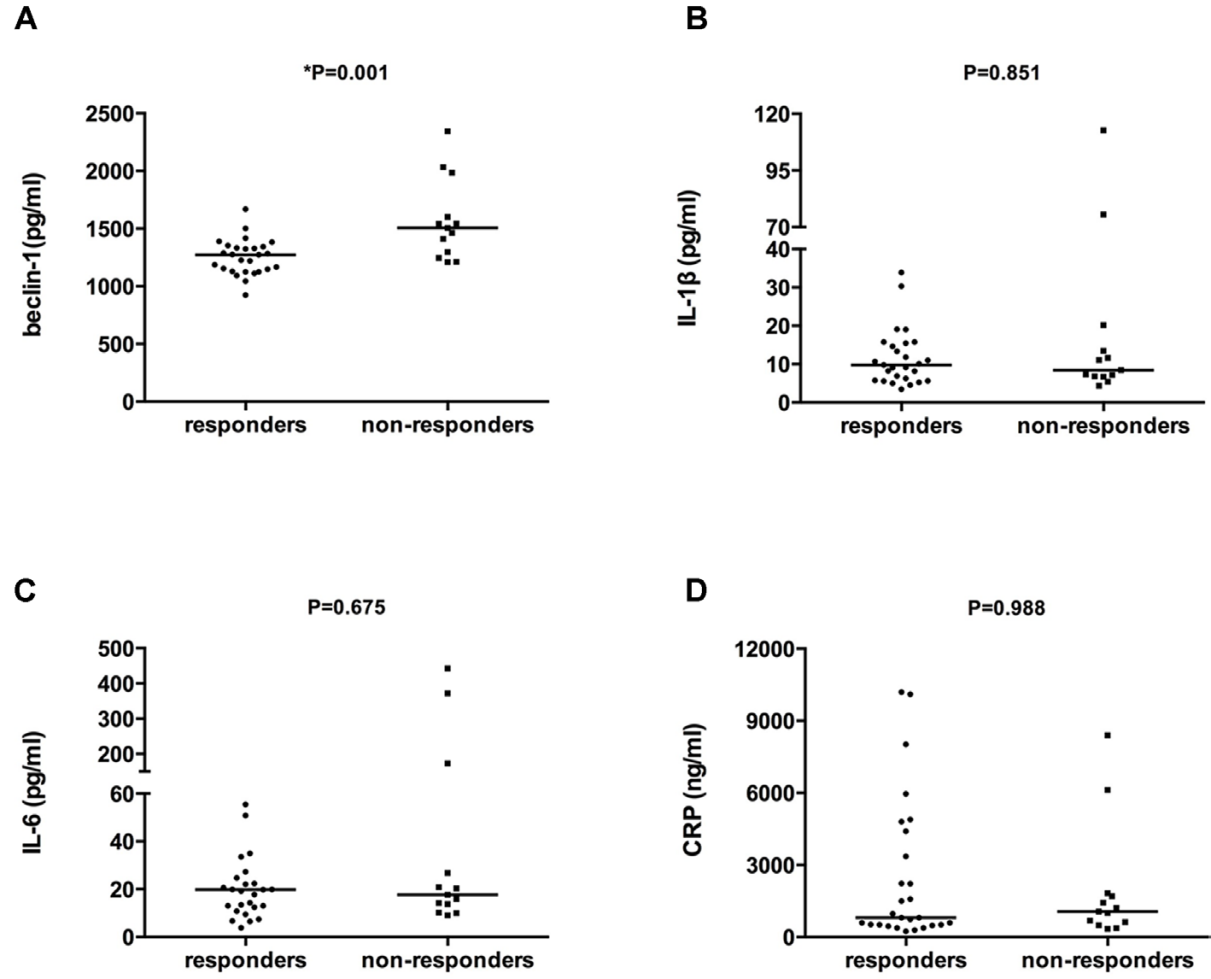
Comparison of serum levels of beclin-1, IL-1β, IL-6, and CRP between antidepressant responders and non-responders at baseline. The bar represents the median. **(A)** Baseline serum beclin-1 levels of responders and non-responders, *p* = 0.001. **(B)** Baseline serum IL-1β levels of responders and non-responders. *p* = 0.851. **(C)** Baseline serum IL-6 levels of responders and non-responders, *p* = 0.675. **(D)** Baseline serum CRP levels of responders and non-responders, *p* = 0.988. All the comparisons between groups were performed using the Mann–Whitney *U* test.

As shown in **Figure 1**, higher serum beclin-1 levels were detected in non-responders than in responders (1,568.85 ± 349.47 vs. 1,252.76 ± 153.68 pg/ml, respectively, *Z* = −3.191, *p* = 0.001). No significant differences in the levels of serum IL-1β, IL-6, or CRP were detected between non-responders and responders (22.39 ± 33.01 vs. 11.64 ± 7.39 pg/ml, 88.25 ± 148.96 vs. 24.85 ± 21.43 pg/ml, 1,942.59 ± 2,452.99 vs. 2,502.46 ± 3,002.56 ng/ml, respectively, all *p* > 0.05). There were no significant correlations between baseline levels of beclin-1, IL-1β, IL-6, and CRP with age, sex, BMI, or baseline HAMD-17 total scores—neither in the total sample nor in the two study groups (data not shown). Moreover, there were no significant correlations between baseline levels of beclin-1 and baseline IL-1β, IL-6, and CRP levels—neither in the total sample nor in the responder and non-responder groups (data not shown).

### Higher Baseline Serum Levels of Beclin-1 Predict Poor Treatment Response

**Figure 2:**
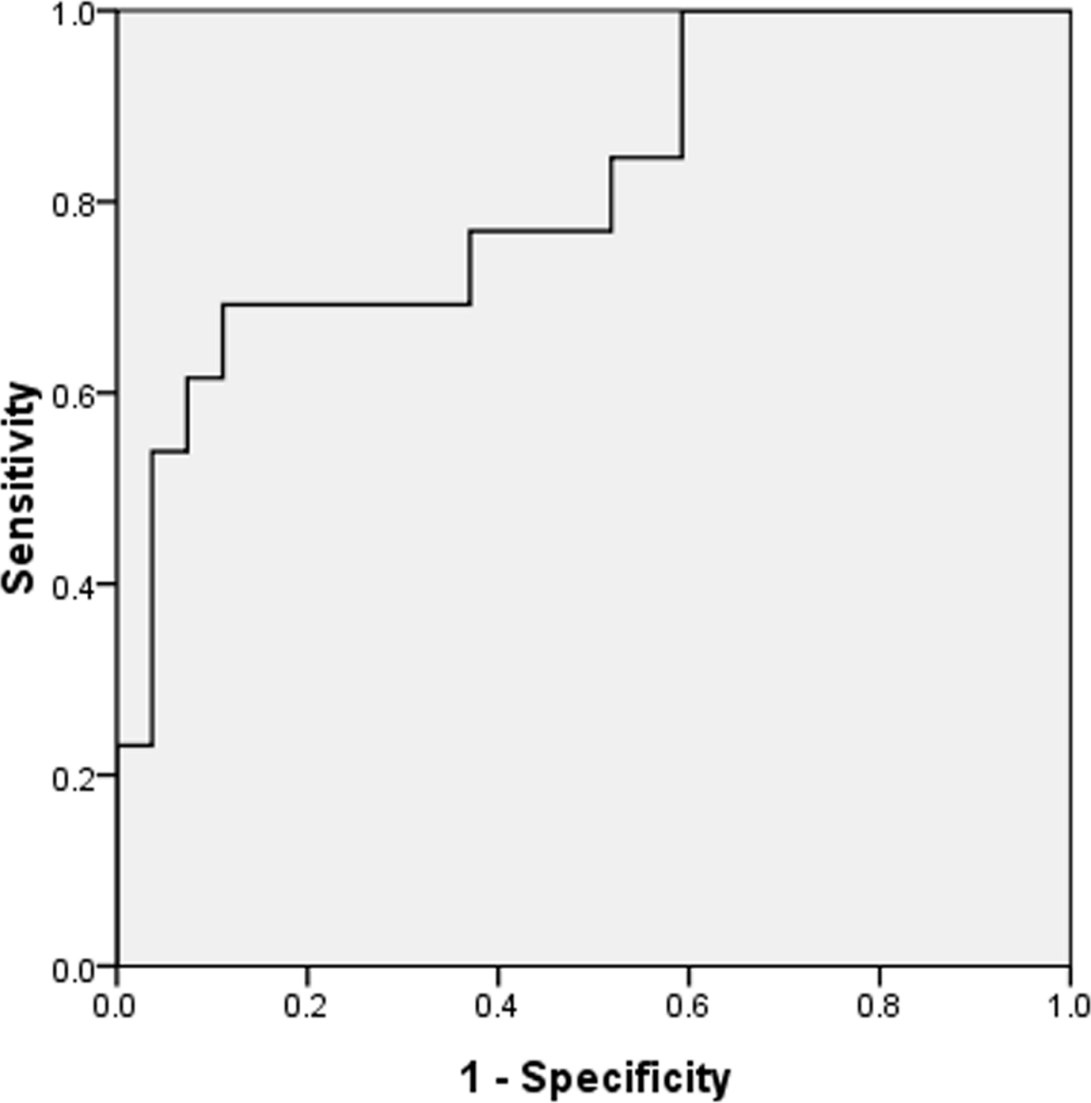
Receiver operating characteristic (ROC) analysis of beclin-1 for distinguishing responders from non-responders. Beclin-1 yields an area under the ROC curve of 0.815 (sensitivity of 69.2% with a specificity of 88.9%).

For each serum biomarker tested at baseline (beclin-1, IL-1β, IL-6, and CRP), univariate logistic regression analysis showed that the beclin-1 level significantly negatively correlated with treatment response [OR = 0.993, 95%  confidence interval (CI) 0.988 to 0.998, *p* = 0.01], while serum IL-1β, IL-6, and CRP level were not significantly correlated with treatment response (all *p* > 0.1) (**Table 2**). Thus, serum IL-1β, IL-6, and CRP were not further investigated in multivariable analysis. We then determined whether beclin-1 level was an independent predictor using two multiple logistic regression models. We found that beclin-1 was the only independent predictor of treatment response after adjusting for potential confounders both in multiple model 1 and model 2 (OR = 0.993, 95% CI 0.988 to 0.998, *p* = 0.01; and OR = 0.993, 95% CI 0.988 to 0.998; *p* = 0.01, respectively) (**Table 3**). There was no multicollinearity among variables included in model 1 and model 2. ROC curve analysis was used to assess the discrimination of the performance of baseline serum beclin-1 levels for the prediction of treatment response. The area under the curve (AUC) of beclin-1 was 0.815 (95% CI 0.669–0.961) (**Figure 2**). The optimal cut-off value for the prediction of treatment response was 1400.68 pg/ml (sensitivity = 69.2% and specificity = 88.9%). Moreover, when baseline serum beclin-1 was entered into the multivariate model as a categorical variable (low or high) using the optimal cut-off value for sensitivity analysis, we found that the baseline serum beclin-1 level was still a significant predictor of treatment response (OR = 0.056, 95% CI 0.01 to 0.299, *p* = 0.001) (**Table 4**). Taken together, these results indicated that beclin-1 may be an independent biomarker for predicting treatment response.

**Table 2:** The results of univariate logistic regression to predict treatment response.

Variable	Odds ratio (95% CI)	*p*-value
Baseline beclin-1 (pg/ml)	0.993 (0.988–0.998)	0.01*
Baseline IL-1β (pg/ml)	0.972 (0.931–1.014)	0.182
Baseline IL-6 (pg/ml)	0.990 (0.977–1.003)	0.141
Baseline CRP (ng/ml)	1.000 (1.000–1.000)	0.554

(95% CI): 95% confidence interval. (OR): odds ratio. *p < 0.05.

**Table 3:** The results of multiple logistic regression to predict treatment response.

	Independent variable	Odds ratio (95% CI)	*p*-value
Multiple model 1	Baseline beclin-1	0.993 (0.988–0.998)	0.01*
	Age	–	0.360
	Sex	–	0.706
	BMI	–	0.773
Multiple model 2	Baseline beclin-1	0.993 (0.988–0.998)	0.01*
	Age	–	0.360
	Sex	–	0.706
	BMI	–	0.773
	Medication (SSRI/SNRI)	–	0.902

Dependent variable: treatment response (0 = no, 1 = yes). Independent variable: model 1 included baseline serum beclin-1 and sociodemographic data (age, sex, and BMI). Model 2 was adjusted for all variables in model 1 and type of antidepressant (SSRI/SNRI). (95% CI), 95% confidence interval; (OR), odds ratio. *p < 0.05.

**Table 4:** The results of multiple logistic regression with optimal cut-off value of baseline serum beclin-1.

Variable	Odds ratio (95% CI)	*p*-value
Baseline beclin-1 (low/high)	0.056 (0.01–0.299)	0.001*
Age	–	0.382
Sex	–	0.877
BMI	–	0.710

(95% CI): 95% confidence interval. (OR): odds ratio. *p < 0.05.

The optimal cut-off value is 1400.68 pg/ml. Low serum beclin-1 was defined as <1,400.68 pg/ml (n = 28). High serum beclin-1 was defined as ≥1,400.68 pg/ml (n = 12).

### Ranking of Input Variables in the Random Forest Model to Predict Treatment Response

**Figure 3:**
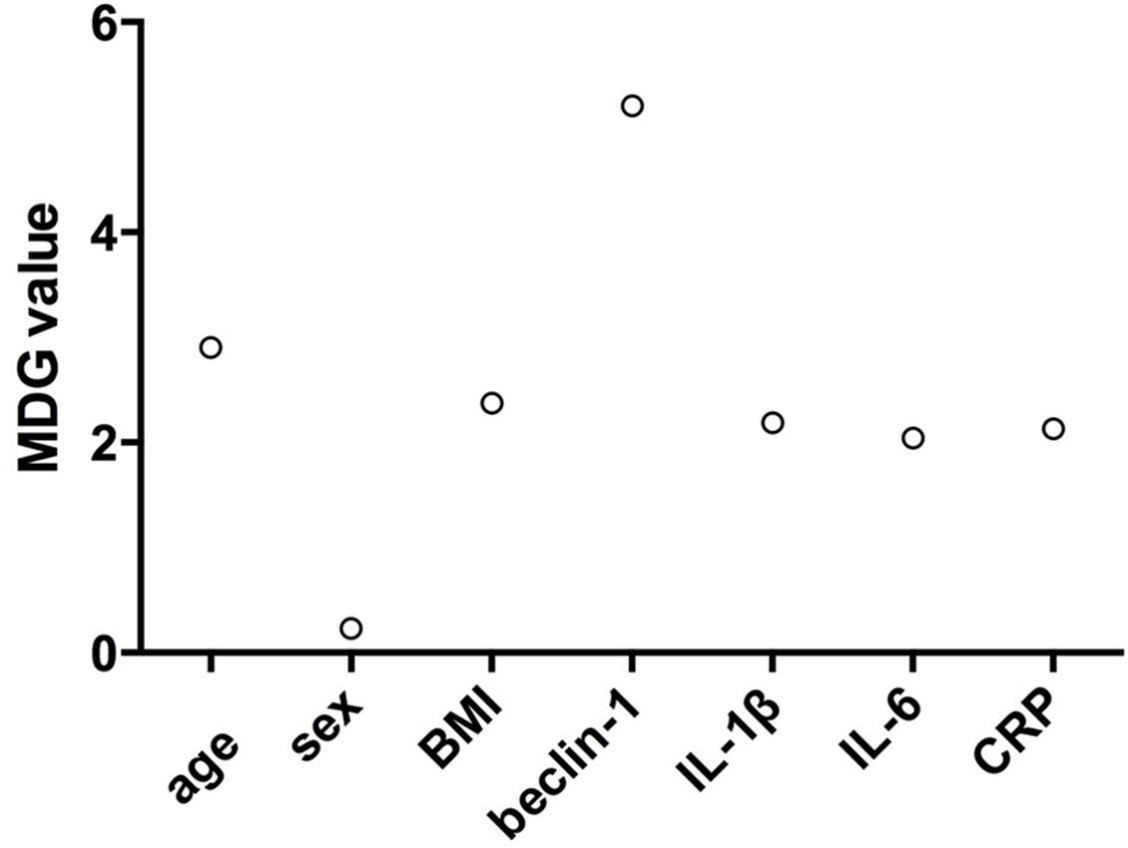
Random forest model was used to sort the order of the importance of variables including four serum factors and demographic data (age, sex, and BMI) for their contribution to predicting treatment response. MDG: mean decrease in Gini.

To further support our results, we used a random forest model to sort the order of importance of these serum-biomarker and demographic data in terms of their contribution to the treatment response. As shown in **Figure 3**, beclin-1 was ranked above all other variables, suggesting that it had higher prediction potential than other variables in our cohort. Based on these results, it appears that serum beclin-1 level is the most important predictor of responses to antidepressant treatment.

### Change in Serum Beclin-1 Levels After Treatment

We compared pre-treatment and post-treatment of serum beclin-1 levels to determine whether antidepressant treatment could change serum beclin-1 levels. No significant change in serum beclin-1 was observed in all patients after 8 weeks of antidepressant treatment (1,355.49 ± 275.33 vs. 1,421.35 ± 326.28, *Z* = −1.290, *p* = 0.197). Further sub-group analysis showed that the serum levels of beclin-1 were significantly increased in the responders group whereas no change was detected in the non-responder group (non-responders: 1,568.85 ± 349.47 at baseline vs. 1,494.26 ± 330.40 at 8 weeks, *Z* = −1.223, *p* = 0.221; responder: 1,252.76 ± 153.68 at baseline vs. 1,386.25 ± 324.60 at 8 weeks, *Z* = −2.210, *p* = 0.027). No significant correlation was found between change in serum beclin-1 level and reduction in HAMD-17 scores in all patients (*r* = 0.309, *p* = 0.053) or each patient subgroup (non-responders: *r* = 0.272, *p* = 0.368; responder: *r* = 0.022, *p* = 0.914) following treatment.

## Discussion

To our knowledge, this is the first study to investigate the relationship between baseline serum beclin-1 and antidepressant response in patients with MDD. In this study, we found that baseline serum beclin-1 levels were significantly higher in non-responders* than in responders*. Moreover, logistic regression models showed that baseline serum beclin-1, not inflammatory factors, was an independent predictor for antidepressant treatment response after adjusting for confounders. Interestingly, baseline serum beclin-1 level showed a significant negative correlation with the therapeutic effect. A higher baseline serum beclin-1 level indicated a worse response to antidepressant medication after 8 weeks of treatment, while lower baseline serum beclin-1 predicted a better treatment response. ROC curve analysis showed that serum level of beclin-1 had a sensitivity of 69.2% and specificity of 88.9% for distinguishing responders from non-responders with an AUC of 0.815. Furthermore, random forest analysis showed serum beclin-1 was the most important predictor for antidepressant response according to the MDG index. Based on all these results, the present study suggests that baseline serum beclin-1 may be used as an independent predictor for response to SSRI or SNRIs in drug-naive or drug-free patients with MDD.

To date, only very few studies have investigated the relationship between beclin-1 expression level and antidepressant response. The only previous study by Gassen et al. reported that beclin-1 expression levels in PBMCs from patients with a current depressive episode at admission showed statistically significant positive correlations with the clinical response after 6 weeks of treatment (11). Our findings are not consistent with their results. The discrepancies between their and our results may also be explained by the difference in clinical characteristics and diagnoses, types of antidepressant drugs, and duration of antidepressant treatment. Why beclin-1 levels may predict antidepressant response is still unknown. It will be interesting for future studies to clarify the biological mechanisms.

Recent reports have indicated that the autophagy pathway is crucially involved in the effects of antidepressants (35). Several studies have reported that antidepressants can increase the expression of autophagy marker, beclin-1, in neural cells and animal models of depression (10, 11, 36). Moreover, a previous study by Gassen et al. reported that PBMCs were collected from inpatients, cultivated, and treated with antidepressants ex vivo for 48 h. They found that antidepressants can increase beclin-1 levels in treated PBMCs and observed a correlation between therapeutic outcome and increase in beclin-1 levels (11).

To date, no study has investigated the effect of antidepressants on beclin-1 levels in patients *in vivo*. Our study is the first to determine and compare the pre-treatment and post-treatment beclin-1 serum levels in patients with MDD. We found that serum beclin-1 levels increased in responders but not in non-responders. Our data provide preliminary evidence that an antidepressant-induced increase in beclin-1 levels is more prominent in responders than non-responders or even exclusively restricted to responders. Thus, our results support the hypothesis that autophagy is a potential mechanism of antidepressant action. Notably, previous *in vitro* studies have suggested that amitriptyline (a tricyclic antidepressant) and citalopram (an SSRI) could increase the expression of beclin-1 in rat primary astrocytes, but venlafaxine fails to exert these effects. Thus, some but not all antidepressants could affect autophagy (10). However, we did not conduct a subgroup analysis based on the type of antidepressants as our sample size was very limited. Further research is needed to clarify the differences in the effect of different antidepressants on autophagy.

In this study, it sounds contradictory that serum beclin-1 levels were higher in non-responders and that antidepressant treatment increased beclin-1 levels in responders. These contradictory findings indicate that the role of beclin-1 in major depression and action of antidepressants are rather complex. In general, autophagy is an adaptive protective mechanism (37). Moreover, autophagy is a beneficial, cell-protective process against neuronal cell death (38). However, excessive autophagy has been shown to be pathogenic (39). For example, excessive autophagy has also been linked to cell death under certain circumstances (40). On the other hand, previous animal and cell studies have showed that beclin-1 may be involved in the effect of antidepressants on autophagy and behavior (11). Based on these results, we speculate that beclin-1 and antidepressants may need to work together to maintain beclin-1 levels at a moderate level. This may be important to the success of the antidepressants. Non-responders may exhibit the condition of having “too much autophagy.” Thus, it is possible that non-responders did not respond to the treatment because they already have high beclin-1 levels. However, the exact mechanism is still far from being understood. It should be further investigated in future studies.

Whether inflammatory factors serve as differential predictors of clinical outcomes in depression therapy remains controversial. Some studies have reported baseline peripheral IL-1β, CRP, and IL-6 levels could be used as predictive biomarkers for antidepressant response (17–19), while other studies do not confirm these results (41–43). In this study, we do not find a significant relationship between baseline serum IL-6, IL-1β, and CRP levels and antidepressant response. The discrepancies between their and our results may be accounted for by the difference in ethnic, demographic, and clinical characteristics of the patients; types of antidepressant drugs; and tissue-specific expression pattern (e.g., serum/plasma). Although our results do not support that baseline serum pro-inflammatory factors could be used as a predictor for SSRIs or SNRI response, additional studies are necessary to confirm such a conclusion as our sample size is small.

Some limitations of this study need consideration. First, this study is a preliminary study, and the sample size was relatively small. Second, different types of antidepressants were used to treat patients, and we did not conduct a subgroup analysis according to the types of antidepressants because of a small sample size. However, our multiple logistic regression analysis showed that baseline serum beclin-1 may predict antidepressant response regardless of drug type (SSRI or SNRI). In other words, serum beclin-1 may not be used to predict response to a specific type of antidepressants. Thus, beclin-1 may not be used to guide the choice of antidepressants but rather to identify patients that potentially may be helped by early adjuvant therapies. Future studies with a large sample sizes are needed to further clarify whether there could be a differential effect of pre-treatment beclin-1 levels on treatment outcomes with SSRI vs. SNRI. Third, we have not investigated potential biological mechanisms underlying the association of low serum beclin-1 level with a good response. The exact mechanism still needs further study. Fourth, whether beclin-1 could change after 8 weeks of antidepressant treatment in other populations and the time taken for beclin-1 levels to change remain unknown. Further studies focusing on other populations are required. Fifth, other autophagy markers, such as LC3B-II/I and p62, have not been systematically investigated in this study. It is necessary to study other markers in the future. Finally, the cellular source of serum beclin-1 remains unclear, and whether the serum levels of beclin-1 reflect the real levels in the CNS cannot be concluded from the present study. In conclusion, despite the aforementioned limitations, the findings of our study suggest that baseline serum beclin-1 levels may be used as a biomarker to predict antidepressant response in patients with MDD. Moreover, our results provide further support that beclin-1 may be involved in the therapeutic effect of antidepressant drugs.

## Ethics Statement

The protocol of this study was approved by the Ethics Committee of the Shang Hai Mental Health Center. All patients gave informed consent for the biomarker study.

## Author Contributions

Author SH performed the statistical analyses and wrote the manuscript. Author DZ and ZL provided assistance for the laboratory work. Author JS completed all of the data entry. Authors FX, NZ and QW were responsible for the diagnosis and clinical assessment of the participants. Author HL designed and wrote the study protocol and reviewed the manuscript. In addition, author JZ and WY offered many constructive opinions on this study and provided a critical revision of the manuscript for important intellectual content. All authors contributed to and approved the final manuscript.

## Funding

This study was supported from the Collaborative Innovation Center for Translational Medicine at Shanghai Jiao Tong University School of Medicine (TM201506 and TM201624); Clinical Research Center, Shanghai Jiao Tong University School of Medicine (DLY201620); Youth Scientific Research Project of Shanghai Municipal Commission of Health and Family Planning (20184Y0310); and National Major Project for IND (2018ZX09734005).

## Conflict of Interest Statement

The authors declare that the research was conducted in the absence of any commercial or financial relationships that could be construed as a potential conflict of interest.
